# Development and preliminary validation of a brief nurses’ perceived professional benefit questionnaire (NPPBQ)

**DOI:** 10.1186/s12874-020-0908-4

**Published:** 2020-01-30

**Authors:** Yanli Hu, Jing Hu, Liping Li, Bin Zhao, Xiaohong Liu, Fan Li

**Affiliations:** 1 School of Nursing, Jilin University, Changchun, China; 2 School of Nursing, Naval Medical University, Shanghai, China; 3 School of Nursing, Shanghai University of Traditional Chinese Medicine, Shanghai, China; 4 Department of Pathogenobiology, the Key Laboratory of Zoonosis Research, Chinese Ministry of Education, College of Basic Medicine, Jilin University, Changchun, China; 5 The Key Laboratory for Bionics Engineering, Ministry of Education, Jilin University, Changchun, China; 6 College of Basic Medical Science, Jilin University, No.126 Xinmin Street, Changchun, 130021 China

**Keywords:** Nurses, Psychometrics, Nurses’ perceived professional benefit, Instrument development

## Abstract

**Background:**

With the increased empirical interest in the positive significance of improving nurses’ sense of professional benefits, there is a requirement for measures of nurses’ perceived professional benefit (NPPB). Our objective was to develop and psychometrically test a brief Nurses’ Perceived Professional Benefit Questionnaire (NPPBQ).

**Methods:**

After expert consultation and nurse interviews, a primary questionnaire was developed for an exploratory factor analysis (EFA). The seventeen items of the NPPBQ were used for verification of the theorized factor structure and content validity using a confirmatory factor analysis (CFA). The NPPBQ’s concurrent validity was evaluated. Three samples of nurses were collected in Shanghai, Hangzhou and Nanjing between November 2017 and August 2018.

**Results:**

The results of the EFA and CFA verified the five dimensions of nurses’ occupational benefit discovery. The results demonstrated that the NPPBQ has adequate internal consistency and is fully consistent with the theorized factor structure. This 5-factor solution explained an adequate percentage of the total variance. The Cronbach’s alpha of each dimension of the NPPBQ was good. The concurrent validity was significantly correlated with all aspects of the Maslach Burnout Inventory (MBI).

**Conclusion:**

The results suggest that the NPPBQ is a psychometrically sound measure for evaluating perceived professional benefits among a wide range of nurses.

## Background

Nurses’ perceived professional benefits (NPPB) refer to nurses’ perceptions of the gains and benefits they receive in their profession in the process of employment and the belief that engaging in the nursing profession can promote their all-round growth and development [1, 2]. An appropriate sense of professional gain makes significant contributions to the improvement of job satisfaction and the will to stay of nurses and the retention of nursing staff [3, 4] but is poorly understood from the perspective of the nurses themselves because nursing and nurses were long given a relatively low social and economic status due to the nature of their work, cultural perceptions of social status, income, and gender [5, 6]. With the prevalence of the NPPB and increased empirical interest in the positive significance of improving nurses’ sense of professional benefits, there is a requirement for measures of NPPB that are simple, short (including as few items as possible) and more applicable and practical while evaluating the construct comprehensively.

In a review of the currently utilized and available measures of NPPB published from 1999 to 2019, four articles [7–10] were identified, but only one study [10] involved general registered nurses. The 33-item NPPB questionnaire (NPPBQ) [10] revealed some issues to be resolved. The study in which the scale was developed was conducted using a homogenous Chinese sample from only one hospital, and the questionnaire was never verified by a CFA. Thus, the reliability and validity of the questionnaire need to be further studied. Moreover, considering the busy nature of nurses’ work, the questionnaire, which has 33 items reflecting 5 factors, is not concise enough. Although the literature review identified the use of one tool to assess occupational benefit, this tool only measured nurse preceptors [7, 9]. Preceptors are mainly responsible for providing students with skills and reality-based learning experiences and helping newly hired nurses become familiar with hospital policies, clinical settings, and practical routines. However, preceptors’ perceived professional benefits do not represent the general condition of nurses. Therefore, the development of a reliable and valid tool for assessing NPPB is necessary and meaningful for practice and further studies. To address the above issues, the current study aimed to develop and test a relevant composite NPPBQ that measures nurses’ perceptions of the benefits and rewards they receive from their nursing career and to evaluate its reliability and validity.

The aims of this study were to 1) develop a psychometrically sound instrument that measures nurses’ perceptions of their professional benefits; 2) determine whether the internal structure of this scale is consistent with a consistent conceptual framework using an EFA and a CFA; and 3) determine the initial psychometric properties of the instrument, i.e., internal reliability (Cronbach’s alpha), construct validity and content validity.

## Methods

### Participants

Participants were recruited from seven hospitals with registered nurses. The surveys were conducted by the authors of the present study in Shanghai, Hangzhou, and Nanjing, China (samples 1–2; ZhB), and in Shanghai (sample 3; HJ). Participants in sample 1 were invited to complete the initial 33-item NPPBQ; participants in sample 2 were invited to complete the final 17-item NPPBQ; and participants in sample 3 were invited to complete the 17-item NPPBQ and the Maslach Burnout Inventory (MBI) to evaluate the concurrent validity of the NPPBQ. Participants in samples 1–3 provided written informed consent to complete the 33-item NPPBQ, the 17-item NPPBQ, and the MBI and to supply their demographic characteristics (gender, years working, education, and marital status). Data for three samples were shared with all authors of the present study through data use agreements. The detailed demographic characteristics are listed in Table 1.

**Table 1:** Demographic charactreistics

	Total Sample *N* = 1471	Sample 1 *N* = 588	Sample 2 *N* = 612	Sample 3 *N* = 271
Gender, female n (%)	1456 (99.0%)	580 (98.6%)	609 (99.5%)	267 (98.5%)
Working years, n (%)				
≤ 4	307 (20.9%)	117 (19.9%)	122 (19.9%)	68 (25.1%)
5~9	372 (25.3%)	153 (26.0%)	131 (21.4%)	88 (32.5%)
10~14	332 (22.6%)	122 (20.7%)	143 (23.4%)	67 (24.7%)
15~19	243 (16.5%)	93 (15.8%)	117 (19.1%)	33 (12.2%)
≥ 20	217 (14.8%)	103 (17.5%)	99 (16.2%)	15 (5.5%)
Education, n (%)				
Secondary vocational schools	44 (3.0%)	18 (3.1%)	15 (2.5%)	11 (4.1%)
Junior college	249 (16.9%)	104 (17.7%)	106 (17.3%)	39 (14.4%)
Undergraduate	1156 (78.6%)	456 (77.6%)	481 (78.6%)	219 (80.8%)
Postgraduate or above	22 (1.5%)	10 (1.7%)	10 (1.6%)	2 (0.7%)
Marital status, n (%)				
Married	1031 (70.1%)	424 (72.1%)	461 (75.3%)	146 (53.9%)
Unmarried	424 (28.8%)	159 (27.0%)	146 (23.9%)	119 (43.9%)
Others (divorced or separated)	16 (1.1%)	5 (0.9%)	5 (0.8%)	6 (2.2%)

### Instruments

The survey consisted of a demographic information form, the NPPBQ and the MBI. The MBI [11] is a self-evaluation tool that uses the 7-point Likert rating method. It has a total of 22 items in three domains: emotional exhaustion; depersonalization; and reduced personal accomplishment. The MBI has been translated into a variety of languages and is currently a universal tool for measuring job burnout in various occupational groups. It is also widely used in the field of nursing with high reliability and validity.

### Steps

#### Step 1: item generation

The development of the items on the pilot and final versions of the NPPBQ occurred in four steps described as follows.

##### Concept definition

First, the definition of preceptors’ perceived professional benefits, the 5-dimension NPPB conceptual framework developed by Hu and Liu [12], existing measures, interviews with 23 nurses, and the empirical literature discussing professional benefits or rewards were reviewed and evaluated to provide the definition and to generate items. Considering the impact of understanding and support from the medical team, society, family and patients on the perceived benefits of the nursing profession, NPPB was defined as the gains and benefits nurses perceived that they received from their profession in the process of employment and the belief that engaging in the nursing profession can promote their all-round growth and development. Specifically, five dimensions were distinguished as follows: (1) positive occupational perception; (2) good nurse-patient relationship; (3) recognition from family, relatives, and friends; (4) sense of belonging to a team; and (5), and self-growth (see Additional file 1: Table S1).

##### Initial pool of items

Thereafter, thirty-seven potential items were created to reflect the NPPB construct. The criterion for including an item in the pool of potential items was that the item should fit one of the five potential dimensions (positive occupational perception, good nurse-patient relationship, recognition from others, sense of belonging to a team, and self-growth) and/or relate to the benefits perceived by the nurse as a nurse.

##### Content validity

A panel of experts (independent) and judges (five associate nursing professors, two clinical psychology professors, and 14 clinical nurses with more than five years of experience) discussed, evaluated, and modified these potential items based on the following three principles: (1) whether the items were in accordance with the content of the nurses’ professional benefits; (2) whether the items were suitable and consistent with the NPPB definition and conceptual components; and (3) whether the wording of the items was concise and accurate. Following the above procedure, the first pool of items was reduced to 33 items after the examination and assessment by the judges.

##### Creation of the pilot version

Following the recommendations by Jing Hu and Xiaohong Liu [10], who stated that the measurement of NPPB is more suitable using a five-level Likert scale, 5 response categories ranging from 1 (strongly disagree) to 5 (strongly agree) were used. The following instructions were given: “Nurses’ perceived professional benefits are the gains and benefits that nurses perceive that the profession brings to them in the process of practice and the belief that the nursing profession can promote the overall growth of the self. What benefits and gains do you feel you have experienced from your career? Please read the following statements carefully, consider how well each statement fits your real idea or situation, and click “√” according to your personal experience (1 = Strongly disagree, 2 = Disagree, 3 = Not sure, 4 = Agree, 5 = Strongly agree). There are no “wrong” answers. Please choose only one answer for each statement”.

#### Step 2: item reduction (exploratory factor analysis)

For this step, IBM Statistics *SPSS* version 22.0 was used for the sample 1 data analysis to carry out the preliminary psychometric validation of the NPPBQ. The primary aim of this analysis was to examine the hypothesized factor structure and reduce the 33-item questionnaire to a smaller set of high-performing items to create the final version of the NPPBQ. Therefore, the fixed factor method was used as the criterion to decide the number of dimensions, and an EFA was conducted to test whether the items were consistent with the pre-defined sub-dimensions of the questionnaire (item homogeneity). Based on the results, inconsistent items were removed [13] (items were inconsistent with the pre-defined sub-dimensions; low factor loadings, i.e., < 0.70; low total Cronbach’s alpha if the item was deleted; and low item-questionnaire correlations, i.e., < 0.40), and those items that best reflected the definition and theoretical dimensions of the perceived professional benefits described above were retained. The subsequent items were re-rotated after the removal of items following the factor analysis. Each time an item was deleted, it was re-rotated. The main axis factor rotation method was used for each rotation. Sixteen items were removed during the initial screen, and the items with factor loadings greater than 0.70 were retained. Finally, the 17 items retained in this step were used to create a pilot questionnaire (see Additional file 1: Table S1).

#### Step 3: verification

**Fig. 1:**
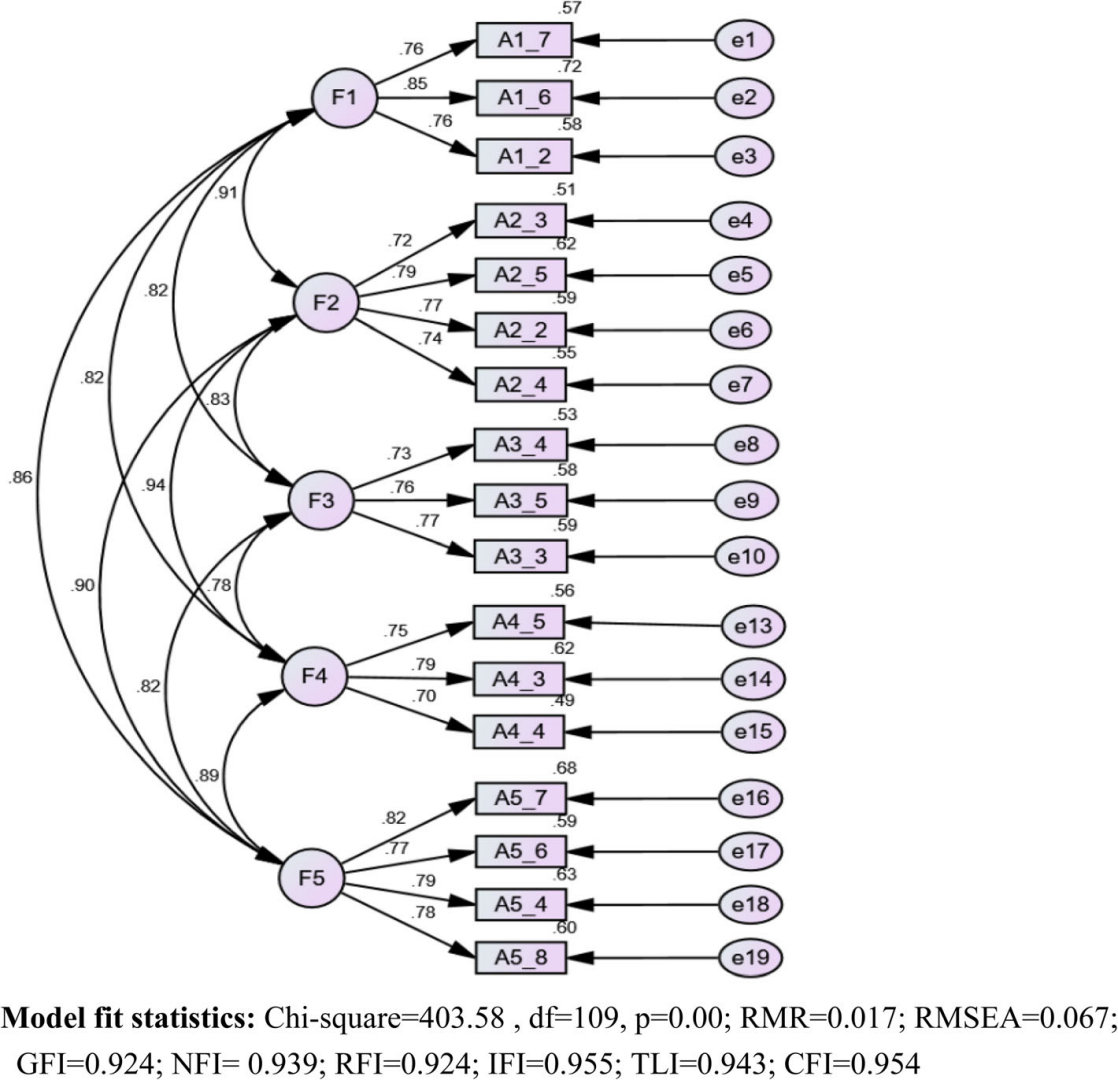
Results from comfirmatory factor analysis models for the five-factor NPPBQ

The analysis in step 3 was conducted to further verify the NPPBQ using a different sample from similar groups. A confirmatory factor analysis with maximum likelihood estimation was conducted to test the underlying factor structure of the NPPBQ. First, the loading values of the first item of each factor were fixed to zero, i.e., the loading values of items A1_7, A2_3, A3_4, A4_5, and A5_7 (see Fig. 1) were set to zero. Second, the inter-factor correlations were not set as fixed zero values because the oblique rotation method was used. There are clear predictions regarding the dimension structure of the measure analyzed. Regarding the CFA analyses, the following multiple indices of fit were considered: the ratio of the χ^2^ to its degrees of freedom (df), the standardized root mean square residual (SRMR), the root mean square error of approximation (RMSEA), the comparative fit index (CFI), the normed fit index (NFI), the Tucker-Lewis index (TLI), and the goodness of fit index (GFI). Amos 24.0 was used to analyze the data of sample 3 in step 3. RMSEA and RMR values of 0.06 or below are indicative of a good fit, and values > 0.06 to 0.08 are considered an acceptable fit. For the GFI, CFI, NFI, and TLI, values ≥0.90 are considered a good fit.

#### Step 4: further validation (concurrent validation)

The primary objective of this step was to assess the stability of the NPPBQ (Guttman split-half coefficient) and to examine its validity in terms of the relationship of the 17-item, five-dimension NPPBQ with the MBI [11]: emotional exhaustion, depersonalization, and reduced personal accomplishment.

## Results

### Sample characteristics

A total of 1447 (out of a possible 1500) nurses completed the survey, for a response rate of 96.5%. The majority of participants were female (*n* = 1456, 99.0%), with a median years worked range of 10–14 years. The majority had an undergraduate education (*n* = 1156, 78.6%) and were married (1031, 70.1%). The sample characteristics are presented in Table 1.

### Questionnaire validation

#### Step 1: item generation

The primary objective of stage 1 was to develop the pilot version of the NPPBQ according to Jing Hu’s five-dimension NPPB model. Thirty-seven items were generated during the initial screening in step 1, and three items were removed after consultation with a panel of experts. The reasons for item deletion were as follows: (a) items were not relevant to the theme of NPPB according to 21 experts’ opinions, (b) items were too similar to other items, and (c) items applied to a minority of respondents (e.g., As a preceptor, clinical teaching work has promoted my continued learning). A total of 33 remaining items were carried forward to step 2.

#### Step 2: exploratory factor analysis (EFA)

The primary aim of step 2 was to further reduce the 33-item questionnaire to a smaller set of high-performing items to create a final version of the NPPBQ. Sixteen items were removed during step 2. The reasons for item deletion were as follows: (a) items were inconsistent with the pre-defined sub-questionnaires, and (b) factor loadings were < 0.70. The KMO (Kaiser-Meyer-Olkin Measure of Sampling Adequacy) index was 0.951, and Bartlett’s Test of Sphericity Approx. Chi-Square was 5826.281 (*P*<0.01). The Principal Axis Factoring method was used for the factor extraction, and the optimal oblique rotation method was used for the rotation because the factors are significantly related. The results of the EFA models shown in Table 2 supported the expected number of factors, with positive occupational perception (with 3 items), good nurse-patient relationship (with 4 items), recognition from family, relatives, and friends (with 3 items), sense of belonging to a team (with 3 items), and self-growth (with 4 items); all items loaded onto the anticipated factor. Five factors explained 60.98% of the total variance (detailed information on the extraction sums of the squared loadings can be seen in Additional file 1: Table S2). All internal consistency coefficients (alpha, a) for the NPPBQ demonstrated acceptable internal consistency [14, 15], with all values above the generally accepted threshold of 0.70. Table 3 shows the means (M), standard deviations (SD), reliability values (Cronbach’s α), and inter-correlations between the NPPBQ’s sub-dimensions and the correlations with the NPPBQ total score. In summary, the step 2 analyses supported the presence of five aspects representing nurses’ sense of the benefits derived from their profession, and all items loaded onto the anticipated factors. Sixteen low-performing items were deleted during step 2, and 17 items were carried forward to step 3.

**Table 2:** The factor loading results of the 17-item NPPBQ

	Component (Pattern Matrix^a^)					Component (Structure Matrix^a^)					Communalities	
	1	2	3	4	5	1	2	3	4	5	Initial	Extraction
A1_7	.850	−.093	.044	−.012	.030	.824	.559	.515	.524	.569	.544	.599
A1_6	.663	.183	−.013	−.023	−.008	.808	.663	.643	.628	.658	.519	.588
A1_2	.595	.030	.181	.046	.074	.766	.627	.494	.526	.554	.495	.571
A2_3	−.071	.863	.066	−.024	−.055	.535	.799	.544	.561	.541	.537	.645
A2_5	.205	.687	−.120	−.103	.095	.660	.788	.558	.685	.639	.441	.533
A2_2	.149	.550	−.038	.204	.011	.623	.748	.456	.525	.575	.464	.475
A2_4	.304	.364	.127	.094	−.129	.615	.642	.532	.541	.495	.565	.578
A3_4	.117	−.145	.736	.065	−.056	.498	.498	.718	.422	.486	.600	.613
A3_5	.120	.040	.700	−.191	.066	.467	.434	.718	.472	.446	.471	.605
A3_3	−.241	.346	.538	.015	.121	.429	.627	.708	.552	.563	.508	.608
A4_5	−.081	−.031	−.160	.817	.164	.558	.584	.550	.773	.574	.424	.526
A4_4	.111	−.007	.074	.736	−.103	.442	.518	.409	.762	.602	.621	.658
A4_3	.045	.052	.227	.416	.118	.572	.622	.636	.720	.648	.603	.675
A5_7	−.161	−.006	.101	.128	.778	.516	.598	.586	.675	.826	.596	.701
A5_6	.157	−.103	.119	−.018	.711	.635	.587	.596	.626	.809	.565	.683
A5_8	.280	.100	−.183	−.001	.604	.655	.613	.445	.592	.750	.551	.616
A5_4	.135	.144	.047	.192	.366	.643	.674	.586	.692	.737	.662	.695

^a^Extraction Method: Principal Axis Factoring. Rotation Method: Promax with Kaiser Normalization

**Table 3:** Means(M), standard deviation (SD), reliabilities (Cronbach’s α), and inter-correlations between the NPPBQ’s subQuestionnaires and correlations with the NPPBQ total score in step 2 (*n* = 588)

	M (SD)	Cronbach’s α	1.	2.	3.	4.	5.	6.
1. Positive occupational perception	11.90 (2.37)	0.84	–					
2. Good nurse-patient relationship	17.54 (2.30)	0.83	.712^**^	–				
3. Recognition from family, relatives, and friends	12.89 (1.82)	0.74	.579^**^	.592^**^	–			
4. Sense of belonging to a team	13.30 (1.55)	0.79	.610^**^	.671^**^	.573^**^	–		
5. Self-growth	17.48 (2.22)	0.85	.704^**^	.731^**^	.602^**^	.732^**^	–	
6. Total score of the NPPBQ	73.13 (8.75)	0.94	.866^**^	.883^**^	.775^**^	.824^**^	.891^**^	–

**Ρ < 0.01, Correlation is significant at the 0.01 level (2-tailed). Guttman Split-Half coefficient of the total scale and every sub-questionnaire were 0.930, 0.795, 0.826, 0.674, 0.725, 0.877 respectively; and Spearman-Brown coefficient were 0.931, 0.855, 0.833, 0.746, 0.796, 0.878, respectively

#### Step 3: confirmatory factor analysis (CFA)

The factorial structure of the NPPBQ was tested using CFA. The results confirmed and validated the previously proposed hypothesis that the theory-driven model reflecting five correlated factors was the best-fitting model of the NPPBQ. The vast majority of the fit indices proved to be good (see Fig. 1). The relative chi-square (χ2/df) = 3.70; RMR = 0.017; RMSEA = 0.067; GFI = 0.924; NFI = 0.939; RFI = 0.924; IFI = 0.955; TLI = 0.943; and CFI = 0.954. The goodness of fit indices (GFI, NFI, RFI, IFI, TLI, and CFI) were greater than 0.90. All regression coefficients were greater than 0.70 (see Fig. 1), and no modifications to the model were needed, demonstrating that the 5-factor model solution of the NPPBQ is robust across samples and meets the previously specified criteria.

#### Step 4

Table 4 presents the mean scores of the final NPPBQ obtained from sample 3, the Cronbach’s alphas of each sub-questionnaire of the NPPBQ, and the correlations with the MBI. The full correlation matrix summarizing the correlations between all the questionnaires and sub-questionnaires (i.e., the correlations of the two questionnaires’ total scores and the interfactor correlations) is included in Table 4. The correlations between the NPPBQ scores and existing measures of the MBI supported the convergent validity of the questionnaire. All correlations were in the anticipated direction (i.e., there was a negative correlation between all dimensions of the NPPBQ and the emotional exhaustion dimension of the MBI, but there was a positive correlation with the personal achievement dimension).

**Table 4:** Descriptive statistics (means and standard deviation) and correlationa among variables in study 3 (*n* = 271)

	M (*SD*)	1.	2.	3.	4.	5.	6.	7.	8.
1. Total score of the NPPBQ	71.16 (9.42)	α = 0.937							
2. Positive occupational perception	11.47 (2.53)	.863^**^	α = 0.840						
3. Good nurse-patient relationship	17.08 (2.44)	.870^**^	.677^**^	α = 0.828					
4. recognition from family, relatives, and friends	12.74 (1.87)	.776^**^	.575^**^	.582^**^	α = 0.737				
5. sense of belonging to a team	12.86 (1.75)	.846^**^	.641^**^	.691^**^	.586^**^	α = 0.788			
6. self-growth	17.01 (2.40)	.905^**^	.728^**^	.723^**^	.639^**^	.752^**^	α = 0.862		
7. emotional exhaustion	30.13 (10.65)	−.514^**^	−.530^**^	−.407^**^	−.334^**^	−.402^**^	−.491^**^	–	
8. reduced personal accomplishment	38.99 (11.19)	.273^**^	.169^**^	.237^**^	.200^**^	.277^**^	.292^**^	−.076	–
9. depersonalization	10.59 (4.94)	−.366^**^	−.318^**^	−.286^**^	−.290^**^	−.318^**^	−.352^**^	.616^**^	−.171^**^

Pearson’s correlation coefficient test was used, two-tailed. Simplified Chinese version of Palliative Care Spiritual Care Competency Scale (NPPBQ)

**. *Ρ* < 0.01, Correlation is significant at the 0.01 level (2-tailed). Cronbach’s alpha on the diagonal in parenthesis

## Discussion

This paper is the first to report the development and preliminary validation of the brief NPPBQ, which was derived from the conceptual framework established by Hu J. and Liu X. H [12].. The psychometric examinations provided an initial test of the NPPBQ across clinical samples. The four steps provided promising evidence supporting the NPPBQ as a psychometrically sound, factorially stable, and brief measure of NPPB (for the final version of the NPPBQ see Additional file 2: the English version and Chinese version of the NPPBQ).

Across the studies, the factor structure, reliability (Cronbach’s alpha and Guttman split-half reliability), and convergent validity of the NPPBQ were examined. The psychometric analyses of the NPPBQ supported the stability, validity, and good internal consistency of the instrument. The NPPBQ has a brief five-factor structure that showed sound validity and can be described as follows: (1) positive occupational perception; (2) good nurse-patient relationship; (3) recognition from family, relatives, and friends; (4) sense of belonging to a team; and (5) self-growth. The Cronbach’s alphas of all sub-questionnaires of the NPPBQ were 0.84, 0.83, 0.74, 0.79, and 0.85. In addition, the Guttman split-half values were 0.795, 0.826, 0.674, 0.725, and 0.877. The five dimensions revealed good consistency with the components of J. Hu and X. H. Liu’s model and the current literature on NPPB [4, 7–9, 12, 16]. The results suggest that the NPPBQ is theoretically and empirically valid.

The present study provided sufficient evidence supporting the 5-factor NPPBQ model and suggests that the solution is robust across Chinese samples. Additional validation in other cultures is a necessary future research direction. The results suggest that the factor structure for the NPPBQ is stable and clear. The five-dimension structure showed a good fit in terms of both the fit indices (e.g., TLI, CFI, and NFI) and absolute fit indices (e.g., χ^2^/df, RMR, and RMSEA). Importantly, the 5-factor model fits the conceptual framework underlying the NPPBQ [12]. The NPPBQ supports NPPB as a multidimensional construct and the foundational role of cognitive evaluation processes in the generation and maintenance of reasonable vocational cognition and evaluation; it also indicates that NPPB has an intermediate regulating effect on the relationship between job stress and job burnout. The five factors of the NPPBQ represent professional benefits in terms of both the material and non-material benefits of being a nurse. Thus, the NPPBQ significantly contributes by providing a valuable instrument measuring NPPB and assessing the unique aspects of this construct, such as nurses’ perceived nurse-patient relationships and support from important others. Therefore, targeted measures can be taken to improve those relevant aspects.

Concurrent validity analyses found that the NPPBQ was significantly negatively correlated with the emotional exhaustion and depersonalization dimensions’ scores (measured by the MBI). These results were anticipated given the strong relationship between the degree of nursing burnout and NPPB [17, 18]. Thus, the findings further support the validity of the NPPBQ.

According to a previously published study, the sense of occupational benefit can explain 31.6, 13.1, and 9.5% of the variance in emotional exhaustion, depersonalization and reduced personal achievement [17]. Similar to the results of the current study, it has been shown that NPPB is significantly positively associated with reduced personal accomplishment scores on the MBI. It seems plausible that nurses low in NPPB might tend to lack motivation and initiative in their work, resulting in a lower sense of job satisfaction and personal accomplishment. Moreover, a cognitive intervention program for nurses’ sense of occupational benefit can improve the level of professional benefit of nurses and alleviate their burnout [19]. Conversely, a nurse with a higher sense of professional benefit will have a higher sense of personal accomplishment. Accordingly, it is well illustrated that NPPB is associated with self-efficacy and reduced nursing burnout.

In short, a theory-driven measure of NPPB was developed using three samples. The findings of the tests promisingly support the psychometric properties of the NPPBQ in terms of the construct validity and internal consistency. The NPPBQ has five sub-questionnaires. Thus, the NPPBQ reflects important features of NPPB, such as specific career cognition, sense of support from important others and self-development and, moreover, is a brief instrument that assesses all the constructs underlying NPPB as posited by Hu and Liu’s model [12]. Importantly, the NPPBQ is a simple assessment measure. A brief questionnaire with a reasonable number of items will solve the shortcomings of a lengthy questionnaire, such as potential, missingness, and reduced data quality and response burden.

The NPPBQ was developed as a multidimensional instrument to assess registered nurses’ perceived professional benefits. The systematic literature review revealed relatively little research on the development of tools to measure the multiple aspects of professional benefits that nurses perceive. Although there are some related tools, few have focused generally on clinical nurses’ perspectives of their professional benefits, and few have been psychometrically verified with respect to their factor structure. The 5-factor NPPBQ is consistent with a former NPPB conceptual framework and further confirmed it in theory and practice.

### Limitations

As the current study is the first to evaluate the reliability and validity of the 17-item NPPBQ, the present results need to be replicated in other cultural contexts for further validation. There are several areas to be considered for future work. First, an interpretable structure that agrees with the theoretical design that served as the basis for the scale development was provided in the present study, and clinical practicality and applicability [20] are our main aims in developing this NPPBQ; therefore, a five-dimensional questionnaire structure is applied. However, the inter-factor correlations are high (systematically larger than .579), i.e., the results of the present study suggest that the 17-item NPPBQ may be treated as unidimensional [20] at the cost of some loss of information. In addition, single-factor score estimates could possibly be appropriate for a quick or general screening of nurses with low levels of perceived professional benefit (NPPB). Thus, it is possible for the NPPBQ to be regarded as a single dimension, and we aim to further analyze the feasibility of this possibility in subsequent studies. Second, the three samples used in the current study were all samples of convenience, which could limit the generalizability of the results, and evidence suggests that there is a need to conduct subsequent assessments in a large random clinical sample. Third, a test of the factor structure of the NPPBQ using different samples is necessary. Future studies should pursue structural invariance testing via a CFA using diverse populations to determine whether the factor construct of the NPPBQ varies among members of different context groups (e.g., with religious beliefs vs. without religious beliefs). Moreover, the psychometric properties of the NPPBQ should be evaluated across different cultures to further assess its robustness as an evaluation tool. Future studies also should consider performing comparative assessments with existing measures of job satisfaction and nurses’ professional benefits and provide extended evidence of the construct validity of the NPPBQ.

Despite these above limitations, the present study represents the first significant and substantial step toward the evaluation and validation of the NPPBQ. The current study provided promising evidence suggesting that the NPPBQ is a conceptually and empirically valid and reliable tool for the assessment of the dimensions of constructs congruent with the NPPB conceptual model and could be useful to investigators.

### Implications for nursing

There is increasing acknowledgement of the significance and value of guiding nurses to fully realize the benefits of their careers and of improving NPPB in practice. Empirical research has identified significant associations among NPPB, willingness to stay [21], job burnout [21], subjective well-being [22], innovative behavior [23], work engagement [24], professional commitment [25], and sense of calling [26].

The NPPBQ may assist clinical nursing managers in exploring and improving staff perceptions, evaluations, and attitudes towards professional benefits. Findings from the NPPBQ could inform the selection of topics for improving nurses’ good professional perceptions to enhance the assessment of the intensity and influencing factors of NPPB, monitoring changes in practice. Continued and increased facilitation of access to these benefits by medical institutions is vital for sustaining active involvement in the nurse role, and there is a need to improve nurses’ intent to stay against the background of the global shortage of nursing staff and the predicted shortage of nurses.

Another potential use of the NPPBQ would be as an assessment or evaluation tool in education and continuing education departments to inform the content of career development programs and evaluate the effectiveness of such training in bringing about change in attitudes, perceptions and practices.

## Conclusions

While few studies have investigated the professional benefits perceived by nurses in specific specialties, there is no brief questionnaire with good reliability and validity to measure the general condition of most nurses. Given the prevalence of positive psychology, it is important to understand how paying attention to and improving nurses’ sense of career benefit is related to their willingness to stay and can reduce job burnout, which is conducive to improving nurses’ professional mentality and professional identity. The NPPBQ is a multidimensional measure that provides an opportunity to gain further insight and identify approaches to support reasonable professional evaluations of and healthy professional attitudes among nurses.

## Supplementary information

**Additional file 1:** **Table S1.** Items and descriptions of the factors of the NPPBQ. **Table S2.** Total variance explained using PCA.

**Additional file 2.** The English version of the nurses' perceived professional benefit questionnaire (NPPBQ)

## Data Availability

The datasets generated and/or analyzed in the current study are available upon request from the coauthor Yanli Hu in the format of SPSS files.

## Abbreviations

CFA: Confirmatory factor analysis
CFI: Comparative fit index
CVI: Content validity of items
EFA: Exploratory factor analysis
GFI: Goodness of fit index
KMO: Kaiser-Meyer-Olkin measure of sampling adequacy
M: Means
MBI: Maslach Burnout Inventory
NFI: Normed fit index
NPPB: Nurses’ perceived professional benefits
NPPBQ: Nurses’ perceived professional benefit questionnaire
RMSEA: Root mean square error of approximation
SD: Standard deviations
SRMR: Standardized root mean square residual
TLI: Tucker-Lewis index

## References

[1] Hu J, Liu X (2012). Research and thinking on nurses’ perceived professional benefits [J]. Chin J Nurs.

[2] Zhou H, Zhu Y, Zhang X (2018). Psychological capital and perceived professional benefits: testing the mediating role of perceived nursing work environment among Chinese nurses.[J]. J Psychosoc Nurs Ment Health Serv.

[3] Qingqing Z, Yang J, Aihua Z (2017). Relationship between nurses’ professional benefit and job satisfaction and willingness to stay [J]. Chin J Pract Nurs.

[4] Ma HW, Dan X, Xu SH (2015). Nurses’ perceived benefits of trauma nursing rounds (TNR) on clinical practice in an Australian emergency department: a mixed methods study [J]. Australas Emerg Nurs J.

[5] Hollup O (2014). The impact of gender, culture, and sexuality on Mauritian nursing: nursing as a non-gendered occupational identity or masculine field? Qualitative study [J]. Int J Nurs Stud.

[6] Ma H, Dan X, Xu S (2017). Current status of nurses’ perceived professional benefits and influencing factors in 3A-level hospitals in Tianjin [J]. Chin J Ind Hyg Occup Dis.

[7] Usher K, Nolan C, Reser P (1999). An exploration of the preceptor role: preceptors’ perceptions of benefits, rewards, supports and commitment to the preceptor role [J]. J Adv Nurs.

[8] Sharoff L (2008). Exploring nurses? Perceived benefits of utilizing holistic modalities for self and clients [J]. Holist Nurs Pract.

[9] Hyrkäs K, Shoemaker M (2010). Changes in the preceptor role: re-visiting preceptors’ perceptions of benefits, rewards, support and commitment to the role [J]. J Adv Nurs.

[10] Hu J, Liu X (2013). The preparation of nurses’ occupational benefit questionnaire and its reliability and validity test [J]. J Chin People's Liberation Army.

[11] Valente MDSDS, Wang YP, Menezes PR (2018). Structural validity of the Maslach burnout inventory and influence of depressive symptoms in banking workplace: unfastening the occupational conundrum [J]. Psychiatry Res.

[12] Xu J, Liu X (2014). Qualitative research on the conceptual framework of “nurses’ perceived professional benefits” [J]. J Nurses Train.

[13] Hu YL, Tiew LH, Fan L (2019). Psychometric properties of the Chinese version of the spiritual care-giving scale (C-SCGS) in nursing practice [J]. BMC Med Res Methodol.

[14] Agbo A (2010). Cronbach’s alpha: review of limitations and associated recommendations [J]. J Psychol Afr.

[15] Taber Keith S. (2017). The Use of Cronbach’s Alpha When Developing and Reporting Research Instruments in Science Education. Research in Science Education.

[16] Hongzhen Z, Yafang Z, Xiaomei Z (2018). Psychological capital and perceived professional benefits: testing the mediating role of perceived nursing work environment among Chinese nurses [J]. J Psychosoc Nurs Ment Health Serv.

[17] Hu J, Hu X (2016). China Nursing Management, 2014, 14(1): 56-60. Study on the effect of nurses’ occupational benefit on burnout [J]. Nurs Res.

[18] Li N, Zhang R (2016). Correlation between occupational benefit and job burnout of nurses [J]. J Nurs Res.

[19] Mao B, Hu Y, Liu X (2016). Cognitive intervention study on nurses’ sense of occupational benefit [J]. Chin J Nurs.

[20] Garrido CC, González DN, Lorenzo-Seva U, Ferrando PJ (2019). Multidimensional or essentially unidimensional? A multi-faceted factor-analytic approach for assessing the dimensionality of tests and items. Psicothema.

[21] Xiao X, Zhang D, Xiong L (2016). Study on the influence of nurses’ sense of benefit on the retention of employment [J]. China Nurs Manag.

[22] Xiao X, Zhang D, Xiong L (2015). Study on the influence of nurses’ sense of professional benefit on their work input [J]. J Nurs Adm.

[23] Shi J, Song G, Xie L (2016). Study on the relationship between nurses’ professional benefit and subjective well-being [J]. Chin J Nurs Manag.

[24] Gao H, Yang Y, Xu L (2016). Correlation between occupational benefit and innovative behavior of ICU nurses [J]. J Nurs Res.

[25] Chang Hao‐Yuan, Lee I‐Chen, Chu Tsung‐Lan, Liu Ying‐Chen, Liao Yen‐Ni, Teng Ching‐I (2019). The role of professional commitment in improving nurses’ professional capabilities and reducing their intention to leave: Two‐wave surveys. Journal of Advanced Nursing.

[26] Yoon JD, Daley BM, Curlin FA (2017). The association between a sense of calling and physician well-being: a national study of primary care physicians and psychiatrists. Acad Psychiatry.

