# Supplementary material for: Development and preliminary validation of a brief nurses’ perceived professional benefit questionnaire (NPPBQ)
Source: BMC Med Res Methodol. 2020 Jan 30;20:18. doi: 10.1186/s12874-020-0908-4 (PMC6993446; doi:10.1186/s12874-020-0908-4)
Supplement: Supplementary file 1 — Additional file 1: Table S1. Items and descriptions of the factors of the NPPBQ. Table S2. Total variance explained using PCA. [file 12874_2020_908_MOESM1_ESM.doc]

**Supplementary Table 1**

Items and descriptions of the factors of the NPPBQ

| Name of factor | Description | Items |
| --- | --- | --- |
| Positive Occupational Perception | Professional resources, professional advantages, professional image and professional identity (e.g., the nursing profession can provide medical resources, health knowledge, self-care ability and personal resources to the practitioners; being seen as an “angel”, which makes nurse feel proud). This factor mainly reflects that the nurse likes his/her career and is happy that he/she is a nurse. | “I think it is good to be a nurse.”  “The nursing profession allows me to develop my strengths and realize my social value.”  “I am proud of the good image of the nursing profession as being people’s ‘white angel’ and ‘healing the wounded’.” |
| Good Nurse-Patient Relationship | This includes both the nurse's contribution to the patient and the patient's feedback. Refers to the nurse's contribution to the patient mainly in terms of saving the patient's life, promoting the patient's recovery or improvement, disseminating health knowledge to the patient and the patient's understanding of the nurse's work, satisfaction, gratitude, and help and other forms of feedback. | “The praise or gratitude of the patient and his/her family after successfully saving the patient’s life has improved my sense of professional value.”  “The patient improved/healed under my care, giving me a sense of accomplishment.”  “I am very happy to be able to help patients at work.”  “I am pleased that patients can understand my work.” |
| Recognition from Family Members, Relatives, and Friends | This topic refers to the latest and most accurate medical information and quality medical resources that nurses have for their occupations. They can better meet the medical needs and health needs of their relatives and friends. Friends and relatives show support for nurses' work and care for them. The career provides recognition, and this recognition is of great significance to nurses. | “The nursing profession allows me to provide convenient medical resources for my relatives and friends.”  “I can provide professional care when my family is sick.”  “When the family is sick, I can provide them with professional guidance (including medical treatment, medication, etc.).” |
| Sense of Belonging to a Team | Refers to the unity of and mutual assistance provided by the nurse's work team, and the harmonious atmosphere is another occupational benefit that nurses feel. It is mainly reflected in the warmth of the team when the nurses get along well with their work organization, leaders, colleagues, subordinates and students. They are thus eager to belong to and become a member of this group. | “I can get guidance and affirmation from the leadership at work.”  “Mutual communication with colleagues enhances my confidence and strength.”  “My work team can help each other and gets along well, making me feel warm.” |
| Self-Growth | The theme refers to the comprehensive ability of nursing work to train nurses and promote the growth of nurses' professional ability, psychological quality, personality characteristics, life attitude and interpersonal ability. | “I often encounter unexpected situations in my work, which improves my psychological quality (such as coordination, resilience, etc.).”  “The nursing profession has taught me how to deal with various people and enhance my interpersonal skills.”  “As my professional skills continue to improve, my professional mentality is maturing.”  “The nursing profession has made me develop a patient and meticulous style.” |

**Supplementary Table 2**

Total variance explained using PCA.

| **Factor** | **Initial eigenvalues** | | |  | **Extraction sums of squared loadings** | | |  | **Rotation sums of squared loadings** |
| --- | --- | --- | --- | --- | --- | --- | --- | --- | --- |
|  | Total | % of variance | Cumulative % |  | Total | % of variance | Cumulative % |  | Total |
| 1 | 8.736 | 51.390 | 51.390 |  | 8.353 | 49.136 | 49.136 |  | 6.402 |
| 2 | 1.039 | 6.112 | 57.503 |  | .642 | 3.779 | 52.915 |  | 6.737 |
| 3 | 1.017 | 5.981 | 63.484 |  | .596 | 3.506 | 56.421 |  | 5.665 |
| 4 | .826 | 4.861 | 68.345 |  | .460 | 2.708 | 59.129 |  | 6.378 |
| 5 | .696 | 4.093 | 72.438 |  | .315 | 1.854 | 60.983 |  | 6.647 |
