# Supplementary material for: Development and preliminary validation of a brief nurses’ perceived professional benefit questionnaire (NPPBQ)
Source: BMC Med Res Methodol. 2020 Jan 30;20:18. doi: 10.1186/s12874-020-0908-4 (PMC6993446; doi:10.1186/s12874-020-0908-4)
Supplement: Supplementary file 2 — Additional file 2. The English version of the nurses' perceived professional benefit questionnaire (NPPBQ) [file 12874_2020_908_MOESM2_ESM.doc]

The English version of the NPPBQ

**Guidance: *Nurses’ perceived professional benefits* are the gains and benefits that nurses perceive that the profession brings to them in the process of practice and the belief that the nursing profession can promote the overall growth of the self. What benefits and gains do you feel you have experienced from your career? Please read the statements carefully, consider how well each statement relates to you, and click “√” according to your personal experience (1=Strongly disagree, 2=Disagree, 3=****Not sure, 4=****Agree, 5=Strongly agree). There are no “right” or “wrong” answers. Please choose one answer for each statement.**

| **Items** | **Strongly disagree** | **Disagree** | **Not sure** | **Agree** | **Strongly agree** |
| --- | --- | --- | --- | --- | --- |
| 1. I think it is good to be a nurse |  |  |  |  |  |
| 1. The nursing profession allows me to develop my strengths and realize my social value |  |  |  |  |  |
| 1. I am proud of the good image of the nursing profession as being people’s ‘white angel’ and ‘healing the wounded’ |  |  |  |  |  |
| 1. The praise or gratitude of the patient and his/her family after successfully saving the patient’s life has improved my sense of professional value |  |  |  |  |  |
| 1. The patient improved/healed under my care, giving me a sense of accomplishment |  |  |  |  |  |
| 1. I am very happy to be able to help patients at work |  |  |  |  |  |
| 1. I am pleased that patients can understand my work |  |  |  |  |  |
| 1. The nursing profession allows me to provide convenient medical resources for my relatives and friends |  |  |  |  |  |
| 1. I can provide professional care when my family is sick |  |  |  |  |  |
| 1. When the family is sick, I can provide them with professional guidance (including medical treatment, medication, etc. |  |  |  |  |  |
| 1. I can get guidance and affirmation from the leadership at work |  |  |  |  |  |
| 1. Mutual communication with colleagues enhances my confidence and strength |  |  |  |  |  |
| 1. My work team can help each other and gets along well, making me feel warm |  |  |  |  |  |
| 1. I often encounter unexpected situations in my work, which improves my psychological quality (such as coordination, resilience, etc. |  |  |  |  |  |
| 1. The nursing profession has taught me how to deal with various people and enhance my interpersonal skills |  |  |  |  |  |
| 1. As my professional skills continue to improve, my professional mentality is maturing |  |  |  |  |  |
| 1. The nursing profession has made me develop a patient and meticulous style |  |  |  |  |  |

©<<Xiaohong Liu>>: reproduced/translated with kind permission of <<Xiaohong Liu>>
